# The Role of the Notch Signal Pathway in Mucosal Cell Metaplasia in Mouse Acute Otitis Media

**DOI:** 10.1038/s41598-017-04639-z

**Published:** 2017-07-04

**Authors:** Xiang Liu, Ning Cong, Xiang Cheng, Rui Ma, Jing Wang, Yi-bo Huang, Meng Zhao, Xin-wei Wang, Fang-Lu Chi, Dong-Dong Ren

**Affiliations:** 1 Department of Otology & Skull Base Surgery, EYE & ENT Hospital, Fudan University, Shanghai, 200031 P.R. China; 2 Shanghai clinical medical center of hearing medicine, Shanghai, China 200031; 3 ENT Research Institute, EYE & ENT Hospital of Fudan University, Shanghai, 200031 P.R. China

## Abstract

Otitis media (OM) is a major cause of morbidity in pediatric and adult patients. This inflammatory condition is characterized by mucous cell hyperplasia that is thought to produce mucins from the middle ear mucosa. We are interested in the role of Notch signalling pathway in this inflammatory process. Using an acute otitis media (AOM) mouse model through injection of *Streptococcus Pneumoniae* into the middle ear, histopathologic examination and quantitative RT-PCR, acute inflammation with the thickness of mucosa, Goblet cell hyperplasia, and cilia loss were determined and gene expression related to the Notch signaling pathway were evaluated. Upregulation of the mucous cell markers, Argr2 and Muc5AC, and downregulation of the cilia cell marker, Foxj1 and Dnai2, were observed in AOM. In addition, genes encoding Notch receptors and ligands (Notch1, Notch2, Notch3, Notch4 and Dll1) and the Notch target genes (Hes1, Hes5, Hey1, NRARP) in AOM decreased significantly. The expression of the Notch1 and Jagged1 also showed down-regulation throughout the mouse middle ear epithelium. Taken together, this study suggests that downregulation of the Notch signaling pathway is involved in the mucosa hyperplasia during AOM.

## Introduction

Goblet cells are mucus secretory cells that are scattered among the columnar cells near the Eustachian tube orifice in the middle ear epithelium. Their main function is to secrete mucins, forming the mucus layer, which surrounds the cilia and builds up the mucociliary transportation clearance system on the middle ear epithelial surface. Mucus is the first barrier against invaders such as viruses and bacteria^1–3^. Schimada (1971) and Lim (1973) suggested the mucociliary transportation system is present in three distinct tracts. The major tract is the one that starts at the hypotympanum and Eustachian tube pathway; the other two tracts are the epitympanum–Eustachian tube pathway and the minor path from the promontory to the Eustachian tube. Fluid and debris are continuously being cleared from the middle ear cavity to the Eustachian tube by mucociliary clearance^4, 5^. Effusion in the middle ear as a result of infection is commonly seen clinically. Histology can detect thickening of the epithelium and proliferation of mucous cells^6, 7^. However, mucous cell metaplasia disturbs the well-organized mucociliary transport system at the expense of ciliated cells. Little is known about the control of goblet cell proliferation and metaplasia or differentiation.

The Notch system, comprising Notch receptors 1–4 and serrate and delta-type ligands, is one of the key pathways in the progenitor cell signalling network in the intestine^8, 9^. The canonical notch pathway leads to an activation of the target gene Hes1, which is responsible for progenitor cell maintenance, differentiation towards goblet cells, and inhibition of goblet cell development^9, 10^. The role of Notch signalling in proliferation and differentiation in airway epithelial cells has also been actively studied^11^. Rock *et al*.^12^ showed that sustained Notch activation in basal cells promotes their luminal differentiation, primarily toward secretory lineages. Danahay *et al*.^13^ demonstrated that inhibition of Notch2 prevents goblet cell metaplasia in response to diverse stimuli *in vitro* and *in vivo*. Kang *et al*.^14^ showed that goblet cell metaplasia and inflammation were decreased by inhibition of Notch signalling with a gamma secretase inhibitor (DAPT) in an aeroallergen-induced mouse model of asthma-like disease. However, the role of Notch signalling in the middle ear epithelium remains unclear, as does its role in regulating the behaviour of middle ear epithelial cells during infection.

In the current study, we observed the changes of the mucosa and submucosa and the numbers of goblet cells in mouse acute otitis media (AOM) and the expression of the Notch signal in otitis media. Several members of the Notch system were found to be involved in goblet cell metaplasia through Notch1, Notch2, Hes1, Muc5AC, and ARG2. Here, we report that goblet cell metaplasia is indeed linked to the suppression of the Notch signal pathway in mouse otitis media. The aim of this study is to find out ways to restrain mucous cells metaplasia, and to provide theoretical basis for the prevention and treatment of otitis media.

## Results

### Establishment of acute otitis media in the mouse model

**Figure 1:**
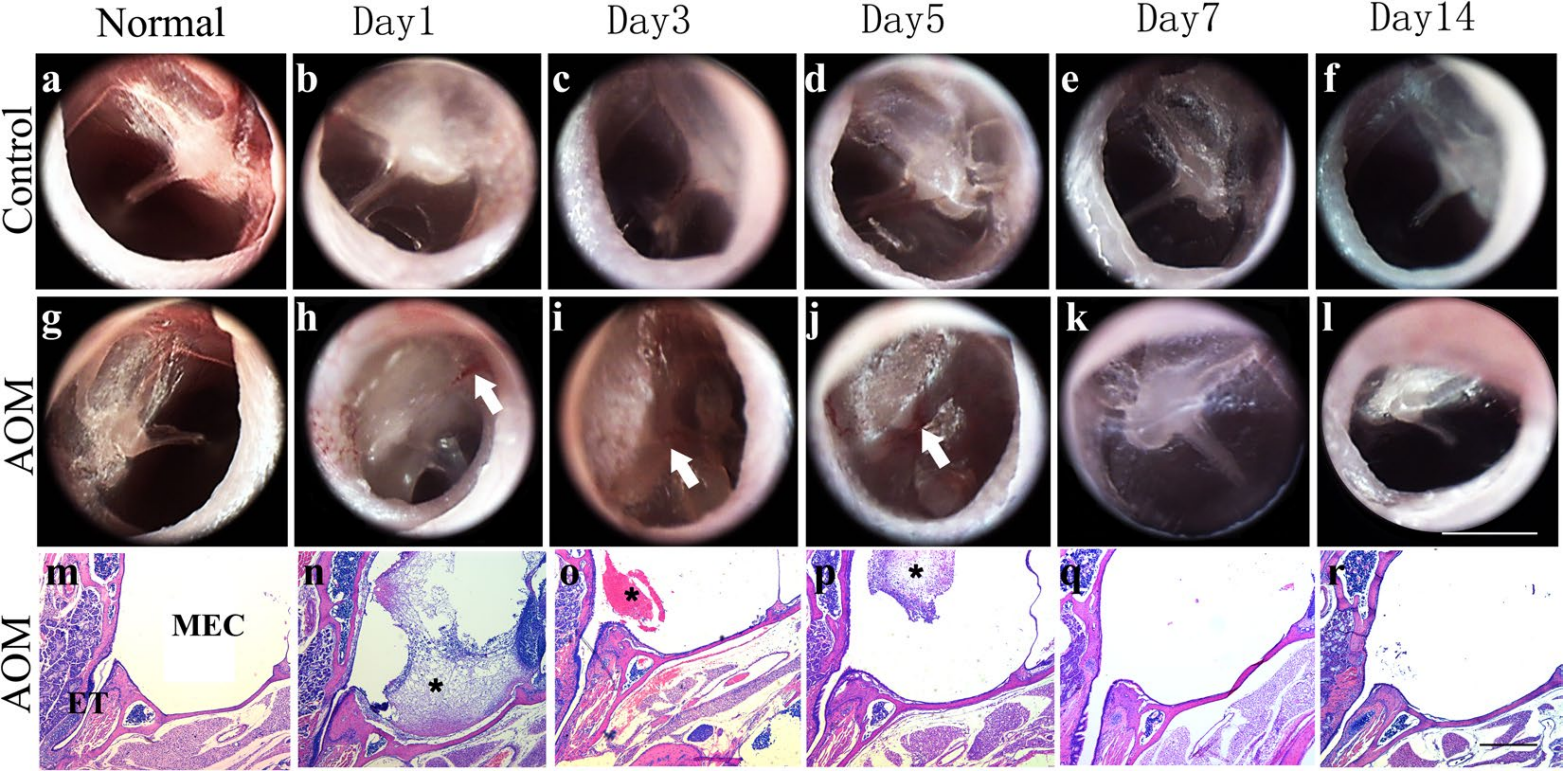
Morphological changes of the tympanic membrane and inflammatory cell infiltration in mouse AOM. Visualization of the tympanic membrane was performed on normal animal and days 1, 2, 3, 7, and 14 post-injection, and photographs were taken at the time of sacrifice under a rigid 4-mm diameter, 0°-angled endoscope. (**a–f**) Tympanic membranes in PBS injected ears as the control group. (**b**) Tympanic membrane thickening was only seen on day 1.Then returned to transparency more quickly and the perforation had healed by days 3–5 (**c**,**d**). With no congestion or oedema seen in control group. (**g–l**) Tympanic membranes in Streptococcus pneumoniae (10^9^cfu/ml, 5 μl) injected ears. (**h**) Tympanic membranes started thickening on day 1. The changes in the tympanic membranes peaked at days 3 and 5 (**i**,**j**). White arrows showed expanded blood vessels. (**k)** All of the pathologic changes began to return to normal starting from day 7. (**l)** On day 14, the membranes were back to normal; in some of them, scars and small calcifications were visible. (**m**–**r)** H&E staining of the bullaes after Streptococcus pneumoniae caused mouse AOM. Inflammatory cells were observed to have accumulated in the middle ear cavity on days 1, 3, and 5 (**n**,**o**,**p**), particularly on day 1. Asterisks show the infiltrated cells. ET, Eustachian tube; MEC, middle ear cavity.

Following *Streptococcus pneumoniae* injection, gross examination of the morphology of the tympanic membrane (TM) was performed by oto-endoscope as shown in Fig. 1a–l). In normal mice, the tensa flaccida of the TM was transparent and thin, and the pars part of the TM was white. One day after *Streptococcus pneumoniae* injection, the tympanic membranes were thickened and whitening (Fig. 1h). Perforations in the membrane could be seen, and some were blocked by secretions. There was no evidence of congestion in the membranes. The changes in the tympanic membranes peaked at days 3 (Fig. 1i) and 5 (Fig. 1j). The entire membrane was still opaque with small congested vessels (Fig. 1, white arrow), and was pink in colour. All of these pathologic changes had begun to back to normal by day 7 (Fig. 1k), at which point most of the perforations in the TM were healed and the membranes were back to transparent. At day 14, the membranes appeared normal; some showed scars and small calcifications (Fig. 1l). In the control group, which was injected with PBS, thickening of the TM could also be seen at day 1 (Fig. 1b), but the TM returned to transparency more quickly and the perforation was healed at days 3 and 5, with no congestion or oedema visible. (Fig. 1c,d)

**Figure 2:**
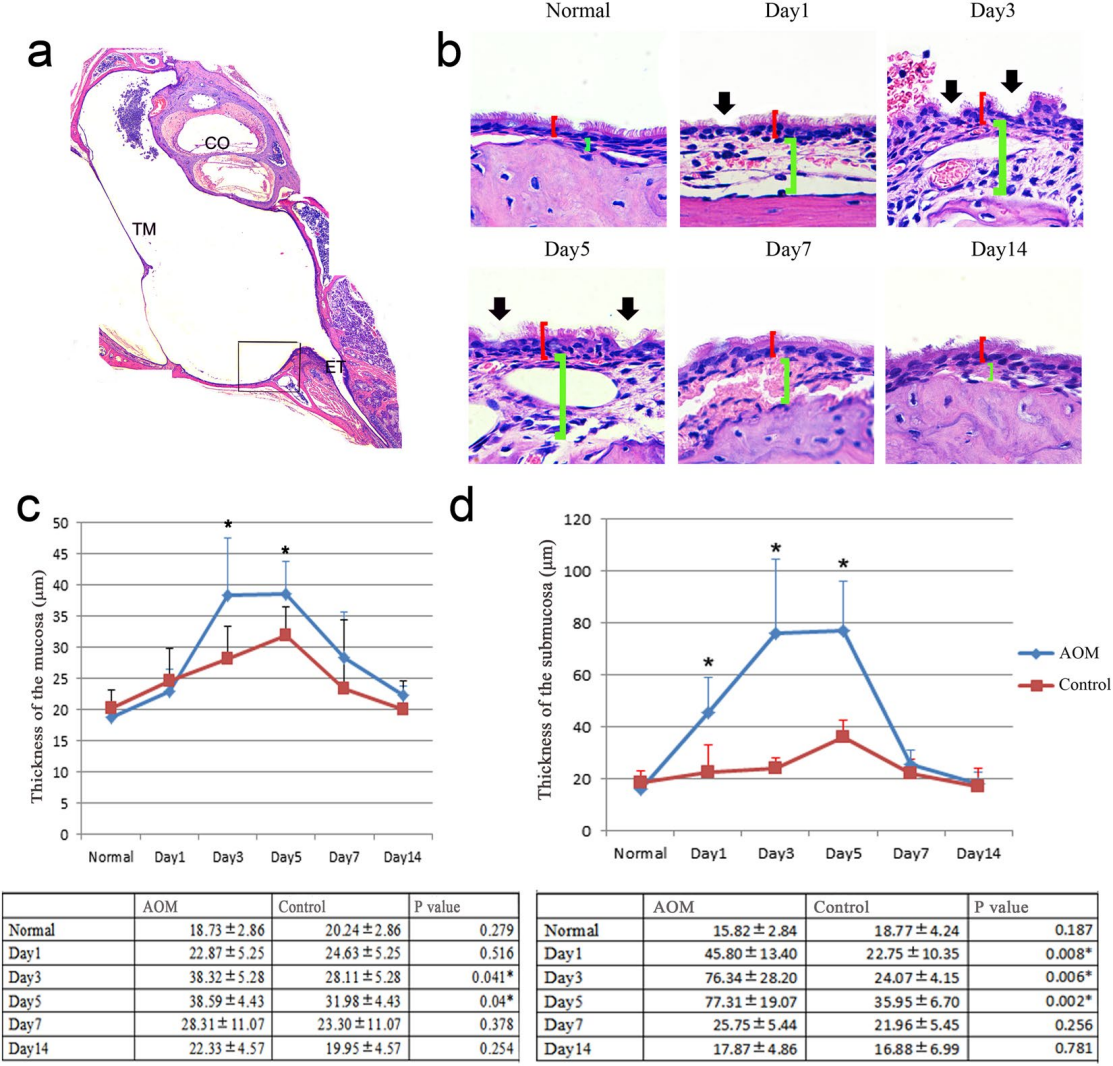
Thickened middle ear mucosa and submucosa in mouse AOM. The same location shown in the box in Fig. 2a in each slide was chosen for measurement. The distance between the apical surface and basement membrane was defined as the thickness of mucosa, and the distance between the basement membrane and the bone surface was defined as the thickness of the submucosa. (n = 6). (**a**) The location of the observation. TM, tympanic membrane; ET, Eustachian tube; CO, cochlea. Scale bar, 500 µm. (**b**) The thickness of the mucosa and submucosa. Red brackets show the thickness of the mucosa; green brackets show the thickness of the submucosa; black arrows show abnormal cilia. Scale bar, 20 µm. (**c**) Average thickness of the mucosa in each group at different times following *Streptococcus pneumoniae* injection. Error bars show S.D. Data are presented as mean + S.D. (n = 6 per group). *p < 0.05. (**d**) The average thickness of the submucosa in each group at different times after *Streptococcus pneumoniae* injection. Error bars show S.D. Data are presented as mean + S.D. (n = 6 per group). *p < 0.05.

There were marked morphological changes in the *Streptococcus pneumoniae*-induced groups compared to controls, including inflammatory cell infiltration, mucosal thickness, and submucosal thickening. Inflammatory cells were accumulated in the middle ear cavity at day 1 (Fig. 1n), day 3 (Fig. 1o), and day 5 (Fig. 1p), most prominent at day 1. The location shown in the box in Fig. 2a, near the Eustachian tube in a similar position in each slide, was used to measure the thickness of the mucosa and submucosa. The middle ear mucosa was thicker after injection of *Streptococcus pneumoniae* than after injection of PBS. In particular, the thickness was significantly greater in inoculated ears on days 3 and 5 compared to controls, as shown in the red brackets in Fig. 2b. The thickness of the mucosa was back to normal from days 7 to 14. The thickness averaged 38.32 μm on day 3 and 38.59 μm at day 5, in contrast to controls, whose thickness averaged 18.73 μm (Fig. 2c). The submucosa of the middle ear was thicker in ears inoculated with *Streptococcus pneumoniae* as compared to controls, as shown in green brackets in Fig. 2b. The increases in thickness were significantly greater between day 1 and day 3 or day 5 than in controls. The average submucosal thickness in the controls was 15.82 μm. In inoculated ears, the submucosa was thickened at day 1 up to 45.8 μm and was much thicker at day 3 and day 5, at 76.34 μm and 77.31 μm, respectively (Fig. 2d).

### Goblet cell proliferation and cilia loss in mouse AOM

**Figure 3:**
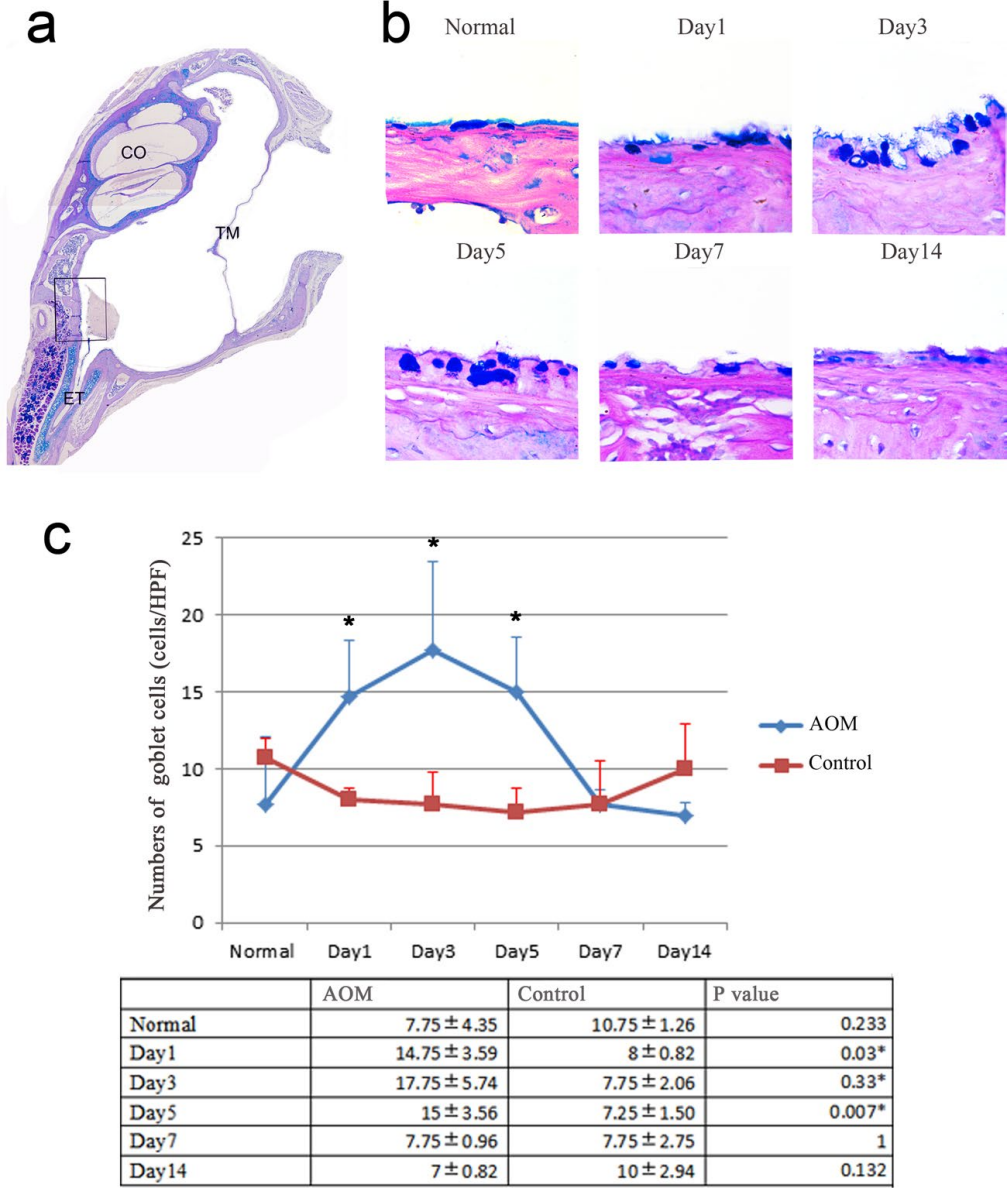
Induction of mucous cell metaplasia (MCM) in the middle ear of mouse AOM. Sections were stained with AB-PAS for evaluation of macroglycoconjugate expression. Goblet cells stained blue or purple in colour by AB-PAS were counted under a light microscope. The same location shown in the box in Fig. 3A in each slide was chosen. The number of goblet cells was counted and expressed as cell number per high-power field. (n = 6). (**a**) The location of observation. TM: tympanic membrane, ET: Eustachian tube, CO: cochlea. (scale bar, 500 µm). (**b**) Increasing AB-PAS positive cells in AOM mucosa compared with control middle ear mucosa. (scale bar, 20 µm). (**c**) The average number of goblet cells per high-power field in each group at different times after *Streptococcus pneumoniae* injection. Error bars show S.D. Data are presented as mean + S.D. (n = 6 per group). *p < 0.05.

Goblet cell numbers were increased after *Streptococcus pneumoniae* injection: there were significant increases on day 1, day 3, and day 5 compared to controls (Fig. 3). The numbers in the control and in inoculated ears on days 1, 3, and 5 were 7.75, 14.75, 17.75, and 15 per high-power field, respectively (Fig. 3c). The number of goblet cells was highest at day 3 and almost back to normal at day 7. As expected, normal cilia architecture was found in healthy epithelium from control subjects. In *Streptococcus pneumoniae*-inoculated epithelia, abnormal cilia architecture was observed, with untidy, shortened, and absent cilia structures as shown in black arrows in Fig. 2b.

**Figure 4:**
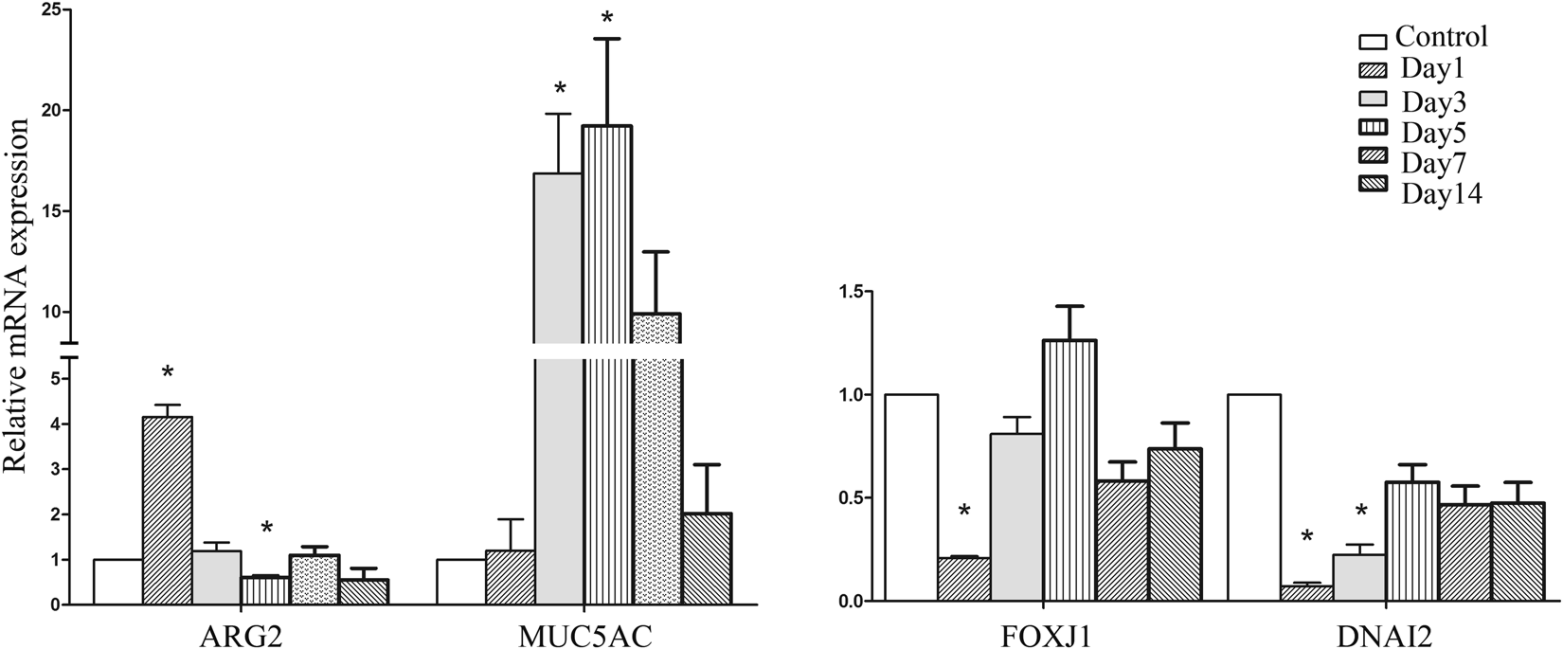
Expression of mucus cell-related genes (MUC5AC, AGR2) and cilia cell-related genes (FOXJ1, DNAI2) in mouse AOM (n = 2, repeated 3 times). MUC5AC mRNA levels were increased at days 3 and 5. ARG2 was increased on day 1. The FOXJ1 and DNAI2 mRNA levels were lower at day 1 than in control subjects.

MUC5AC mRNA levels were dramatically increased at days 3 and 5 compared with control subjects (16.87-fold, 19.22-fold; Fig. 4). The same trend was also observed for ARG 2 (day 1), with 4.16-fold higher expression in inoculated ears over that in control subjects (Fig. 4). These results were in accordance with the AB/PAS staining results that showed goblet cell proliferation. However, mRNA levels in the cilia-related genes FOXJ1 and DNAI2 were decreased at day 1 compared with control subjects (0.21-fold, 0.07-fold; Fig. 4), and DNAI2 mRNA levels were significantly decreased at day 3 compared with the controls.

### Notch signal downregulated in mouse AOM

**Figure 5:**
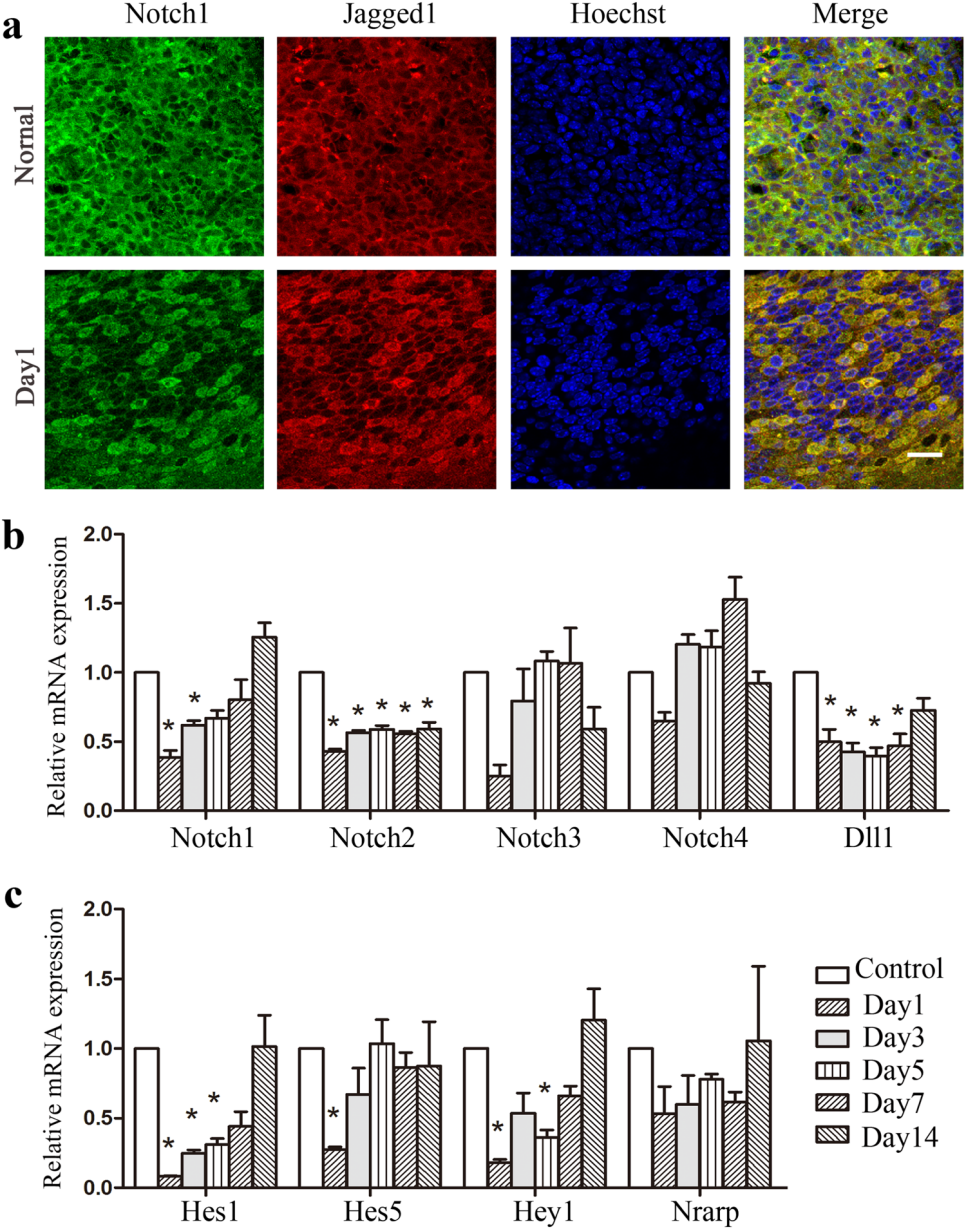
Notch signalling downregulated during AOM. Notch1 and Jagged1 were detected by immunolocalisation. Gene expression of Notch receptors and ligands (Notch1, Notch2, Notch3, Notch4, and Dll1) and Notch signal target genes (Hes1, Hes5, Hey1, and NRARP) was analysed in the mouse middle ear epithelium before and after injection with Streptococcus pneumoniae (n = 2, repeated 3 times). (**a**) Immunostaining of Notch1 and Jagged1 in mouse middle ear epithelium. Faded staining of Notch1 and Jagged1 can be seen at day 1. Scale bar, 20 µm. (**b**) Gene expression of Notch receptors and ligands (Notch1, Notch2, Notch3, Notch4, and Dll1) in mouse AOM. Notch1 mRNA levels were significantly lower on days 1 and 3. Notch2 mRNA levels were greatly decreased through days 1, 3, 5, 7, and 14 in AOM. The Dll1 mRNA levels showed the same trend as Notch2. (**c**) Expression of Notch signal target genes (Hes1, Hes5, Hey1, and NRARP) in mouse AOM. Hes1, Hes5, and Hey1 mRNA expression was significantly decreased at day 1. On days 1, 3, and 5, Hes1 transcript levels were significantly lower than in controls.

As shown in Fig. 5a, intense staining of Notch1 and Jagged1 in the cell membrane was observed co- localized in the majority of cells within the mucosa in normal epithelial cells. Following *Streptococcus pneumoniae* injection, at day 1, faded Notch1 and Jagged1 staining can be seen. The expression of the corresponding genes is shown in Fig. 5b and c. Notch1 mRNA levels were lower in AOM samples on days 1 and 3 than in the controls (0.39-fold, 0.62-fold). The same trend was seen in Notch2 staining; Notch2 mRNA levels were much lower on days 1, 3, 5, 7, and 14 in AOM samples than in controls. Moreover, Dll1 expression showed the same trend as Notch2. However, the expression of Dll1 on day 14 was similar to that in the controls. The mRNA levels of the Notch target genes Hes1, Hes5, and Hey1 (Fig. 5c) were also significantly decreased in AOM at day 1. The levels of Hes1 transcripts on days 1, 3, and 5 were significantly lower than in controls (0.08-fold, 0.25-fold, 0.31-fold). On day 1, Hes5 and Hey1 expression was significantly lower than in controls (0.28-fold, 0.18-fold).

## Discussion

An accumulation of fluid in the middle ear and mucous cell metaplasia-hyperplasia in the middle ear epithelium are the hallmarks of otitis media, especially in otitis media with effusion. It is the histopathologic basis for mucin or mucin-like glycoprotein production in the middle ear cavity, which contributes to the accumulation of middle ear fluid and compromises middle ear function. The factors that modulate mucous cell metaplasia or hyperplasia and mucous secretion in the middle ear cavity are poorly understood. The evidence accumulated to date^15–17^ suggests that initial infection in the middle ear cleft is necessary for the development of otitis media (OM). Infection induces infiltration of inflammatory cells and production of cytokines and chemokines. Cytokines and chemokines, in turn, trigger mucous cell metaplasia (MCM), a classic pathway for triggering mucous cell metaplasia in OM. MCM is induced in acute otitis media but is frequently exacerbated in chronic otitis media^7^.

The results of this study parallel those of other research showing it is possible to establish inflammation in a mouse model with *S. pneumoniae* injections into the middle ear^18, 19^. Use of a certain amount of activated bacteria allowed us to establish an inflammatory response with the typical duration of AOM, yet without systemic complications such as bacteraemia and death. Our data showed that *Streptococcus pneumoniae* induced OM starting at day 1, peaking at days 3–5, and returning to normal at day 14. We examined the most relevant inflammatory measures within the middle ear, monitored changes in the tympanic membrane and performed histological evaluations of the thickening of the mucosa and submucosa. We also confirmed that this AOM model can induce mucous cell metaplasia-hyperplasia, which we evaluated by AB/PAS staining. Along with morphological changes, upregulated mucous cell-related genes (MUC5AC, AGR2) and downregulated cilia-cell related genes (FOXJ1, DNAI2) were also observed. However, the caused is still unknown, as well as whether some classic pathway regulating cell proliferation and differentiation also dominates in mucous cell metaplasia-hyperplasia.

FOXJ1, well known as a regulator of cilia differentiation^20^ and the formation of motile cilia, participates in multiple steps during cilia formation, including centrosome multiplication, docking, and cilia elongation^21^. Downregulation of FOXJ1 by inflammatory factors (e.g., IL-13) can also result in the loss of cilia in the human airway^22^. Consistent with our results, FOXJ1 mRNA levels were lower in epithelium afflicted with OM, and this correlated with the shortness and loss of the cilia that we observed. Secretory MUC5AC mucin is the major structural component of the mucociliary transport system in the Eustachian tube but is not detectable in the middle ear under non-inflamed conditions^23, 24^. However, under inflamed conditions, MUC5AC mucins were highly upregulated in the middle ear in association with an increase in goblet cells (Figs 3 and 4), suggesting a transition from a normal middle ear epithelium to a hypersecretory epithelium.

Notch signalling in the middle ear epithelium appears to be suppressed after infection by *Streptococcus pneumoniae*. The expression of Notch receptors, ligands (Notch1, Notch2, and Dll1), and target genes (Hes1, Hes5 and Hey1) was significantly decreased in AOM, especially on day 1. Notably, the expression of Notch2 decreased throughout the course of infection (Fig. 5b). In our study, the downregulation of the Notch signal was consistent with an enhanced effect of the goblet cell differentiation factors MUC5Ac and ARG2 during inflammation. Other experiments have also shown decreased levels of the Notch signals associated with goblet cell increase^25–27^. van Es *et al*.^25^ showed a rapid, massive conversion of proliferative intestinal crypt cells into goblet cells after conditional removal of the common Notch pathway transcription factor CSL/RBP-J. A similar phenotype was obtained by blocking the Notch cascade with a γ-secretase inhibitor. Our data suggest that the infection led to the suppression of the Notch signal, and then the downregulation of Notch target genes resulted in goblet cell metaplasia. Danahay *et al*.^13^ found that Notch2 acts as a common node downstream of IL-13, as well as the mediators of goblet cell metaplasia. Administration of anti-Notch2 antibodies prevented IL-13 as well as allergen-driven goblet cell metaplasia *in vivo*. However, how the downregulation of the Notch signal controls progenitor cell differentiation into goblet cells in OM needs further investigation.

In this study, we used a mouse AOM induced by injected *Streptococcus pneumoniae* trans-tympanically into the middle ear to observe morphological changes and Notch related gene expression of the epithelium due to AOM. We found opacity and congestion of the tympanic membrane, inflammatory cell infiltration in the middle ear cavity, mucosal and submucosal thickening, increases in goblet cell numbers, and cilia loss and shortening. Most importantly, our data show that downregulation of Notch signalling was correlated with goblet cell metaplasia.

## Methods

### Animals

All experimental procedures and methods, performed strictly under the guidelines and regulations of the Ethical Board of EYE & ENT Hospital of Fudan University, were approved by the Chinese Science Academy Committee on Care and Use of Animals. Adult male BALB/c mice (8 weeks, 20–25 g) were used in this study. All animals were obtained from the Animal Center of the Medical School of Fudan University and were housed under clear conditions. All animals had otoscopic examination prior to experiments to ensure that the external auditory canals and tympanic membranes were normal.

### Middle ear injections

BALB/c mice were anaesthetized using a subcutaneous injection of ketamine (100 mg/ml; 0.067 mg/g body weight) and xylazine (20 mg/ml; 0.013 mg/g body weight). 5 μl of *Streptococcus pneumoniae* type 14 (10^9^cfu/ml) was injected through the tympanic membrane into the right middle ears under a microscope (OPMI 9-FC, Carl Zeiss, Germany) using a 30-gauge needle at the anterior inferior part of the membrane. The Streptococcus pneumoniae type 14 is a gift from Jianghong Xu who bought it from China National Center for Medical Culture Collections (no. 31226)^28^. Left control ears were treated only with trans-tympanic injection of 0.1 M PBS (phosphate-buffered saline, Hyclone) and harvested at the same time points. Mice were sacrificed 1, 3, 5, 7, and 14 days following the injection (n = 6 at each time point). Visualization of the tympanic membrane was performed on days 1, 3, 5, 7, and 14 post-injection, and photographs were taken at the time of sacrifice under a rigid 4-mm diameter, 0°-angled endoscope (Tiansong Brand, Tonglu General Factory of Medical Optical Instruments; Hangzhou City, China).

### Histopathology

For histopathology, the bullae were fixed in a 4% paraformaldehyde solution for 24 h, decalcified with 10% EDTA, and embedded in paraffin. Sections were cut at a thickness of 5–6 μm on the horizontal as previously described^29^ and stained with haematoxylin and eosin for morphologic evaluation. The occurrence of inflammatory cell infiltration was recorded. The thicknesses of the mucosa and submucosa in the inferior tympanum containing the ciliated tract were measured using light microscopy. The distance between the apical surface and basement membrane was defined as the thickness of the epithelium, and the distance between the basement membrane and the bone surface was defined as the thickness of the submucosa. The thicknesses of the epithelium and submucosa were measured using ImageJ software under the microscope in HE-stained sections^30^.

Additional sections were stained with Alcian blue–periodic acid Schiff (AB-PAS) for evaluation of macroglycoconjugate expression. The same areas in each bulla were chosen for measurement of goblet cell numbers. Epithelial cells in the superior ciliated tract stained blue or purple in colour by AB-PAS were identified as goblet cells. AB-PAS positive cells were viewed under a light microscope. The number of goblet cells was counted and expressed as the number of goblet cells per high-power field^23^.

### Immunolocalisation of Notch receptors and their ligands in mouse middle ear epithelium

Whole bullae were removed and fixed in 4% paraformaldehyde for 24 h, and then the middle ear epithelia were dissected out from the bony part of the bullae under a stereomicroscope (Stemi 2000-C; Carl Zeiss, Jena, Germany). The epithelial pieces near the ET orifice were treated with 0.3% Triton X-100 in PBS for 30 min and then blocked using 10% normal donkey serum in PBS for 1 h at room temperature. The tissues were then incubated with the following primary antibodies overnight at 4 °C: goat anti-Jagged1 antibody (diluted 1:200, sc6011; Santa Cruz Biotechnology, Santa Cruz, CA, USA), and rabbit anti-Notch1 antibody (diluted 1:100, D6F11; Cell Signaling Technology; Danvers, MA, USA). After rinsing in PBS, the samples were incubated in fluorescence-labelled secondary antibodies (1:1,000, Alexa Fluor 488 or 555; Invitrogen/Molecular Probes, Carlsbad, CA, USA) at a dilution of 1:1,000 for 2 h at room temperature. For nuclear labelling, samples were additionally incubated with stain (diluted 1:1,000; Invitrogen/Molecular Probes, Carlsbad, CA, USA) for 30 min. After PBS washing, the specimens were mounted on glass slides and examined with a Leica confocal laser-scanning microscope (Leica SP5; Leica Microsystems, Wetzlar, Germany) and images were captured using a 40× objective lens. Images were labelled and spaced using Adobe Photoshop software.

### Middle ear epithelium RNA isolation and quantitative RT-PCR analyses

The middle ear epithelia were dissected out as described above and placed in RNA lysis buffer derived from an RNeasy Micro Kit (74004; Qiagen, Hilden, Germany). After tissue harvest, the middle ear epithelia were homogenized and mRNA extracted as directed by the manufacturer. The mRNAs were subjected to quantitative RT-PCR of transcripts encoding Notch receptors and ligands (Notch1, Notch2, Notch3, Notch4, and Dll1), Notch signal target genes (Hes1, Hes5, Hey1, and NRARP), mucous cell-related genes (Muc5AC, Agr2), and cilia cell-related genes (Foxj1, Dnai2). RNA was quantified using a NanoDrop 2000 (Thermo Fisher Scientific, Wilmington, DE, USA), and all samples were prepared to a concentration of at least 80 ng/μl. Tissues were grouped at each time point for analysis.

Real-time RT-PCR studies were performed using SYBR green reagent and an Applied Biosystems 7500 Real-Time PCR system (Applied Biosystems, Foster City, CA). Total RNA (500 ng) was reverse-transcribed using the PrimerScript RT Master Mix (RR036A, Takara Biotechnology, Japan) as directed by the manufacturer. Then, samples were prepared for RT-PCR using SuperReal PreMix Plus (SYBR Green) (FP205; Tiangen, China). The thermal cycle conditions used were 95 °C for 15 min; 35 cycles of 95 °C for 10 sec, 60 °C for 20 sec, and 72 °C for 32 sec; followed by a melt curve of 95 °C for 15 sec, 60 °C for 1 min, and 95 °C for 15 sec. Data analysis was performed following the manufacturer’s instructions (SABiosciences PCR Array Data Analysis Web Portal). The fold change in gene expression was calculated using the 2−ΔΔCT method^31^ with the aid of the SABiosciences PCR Array Data Analysis Web Portal. The housekeeping control gene used for this method was glyceraldehyde-3-phosphate dehydrogenase. Statistical analyses of the PCR results were performed as previously described^31^.

### Statistical analysis

All experiments were repeated at least twice, and the results were expressed as means. The differences between the control and experimental groups were tested using one-way ANOVA. P <0.05 was considered statistically significant.
